# Experimental Evidence of Epizootic Epitheliotropic Disease Virus (Salmoid Herpesvirus-3, *Alloherpesviridae*) Transmission via Contaminated Fomites and Subsequent Prevention Using a Disinfectant

**DOI:** 10.3390/pathogens10060724

**Published:** 2021-06-09

**Authors:** Mochamad A. Purbayu, Megan A. Shavalier, Mohamed Faisal, Thomas P. Loch

**Affiliations:** 1 Comparative Medicine and Integrative Biology, College of Veterinary Medicine, Michigan State University, 1129 Farm Lane, Room 340G, East Lansing, MI 48824, USA; aji.purbayu@gmail.com (M.A.P.); faisal@msu.edu (M.F.); 2 Department of Fisheries and Wildlife, College of Agriculture and Natural Resources, Michigan State University, 1129 Farm Lane, Room 340G, East Lansing, MI 48824, USA; shavali1@msu.edu; 3 Department of Pathobiology and Diagnostic Investigation, College of Veterinary Medicine, Michigan State University, 1129 Farm Lane, Room 340G, East Lansing, MI 48824, USA

**Keywords:** Salmonid Herpesvirus-3, Epizootic epitheliotropic disease, transmission, disinfection, lake trout, fomite

## Abstract

Epizootic epitheliotropic disease virus (EEDV) has caused considerable mortality in hatchery-reared lake trout *Salvelinus namaycush* in the Great Lakes Basin, and yet the routes of transmission and efficacious means of prevention remain poorly understood. To determine whether EEDV can be transmitted via contaminated fomites and clarify whether such transmission could be prevented via fomite disinfection, juvenile lake trout (n = 20 per treatment) were handled in nets previously soaked in an EEDV suspension (7.29 × 10^4^–2.25 × 10^5^ virus copies/mL of water) that were further immersed in either 1% Virkon^®^ Aquatic (“disinfected” treatment, in triplicate) or in sample diluent (“EEDV-contaminated” treatment). Negative control nets were soaked in sterile sample diluent only. Characteristic gross signs of EED developed in the “EEDV-contaminated” treatment group, which was followed by 80% mortality, whereas no gross signs of disease and 0–5% mortality occurred in the negative control and “disinfected” treatment groups, respectively. EEDV was detected via qPCR in 90% of the “EEDV-contaminated” treatment fish, however, it was not detected in any fish within the negative control or “disinfected” treatment groups. Study findings not only demonstrate that EEDV can be readily transmitted via contaminated fomites, but importantly suggest that Virkon^®^ Aquatic is an efficacious option for preventing EEDV contagion via the disinfection of hatchery tools, thereby highlighting a promising tool for improving lake trout hatchery biosecurity and minimizing EEDV-linked losses.

## 1. Introduction

Since its emergence in the 1980s, Epizootic epitheliotropic disease virus (EEDV; i.e., Salmonid Herpesvirus-3, family *Alloherpesviridae*; [1]) has directly or indirectly killed millions of hatchery-reared lake trout *Salvelinus namaycush* [2] and continues to impede hatchery-based conservation efforts of this invaluable, indigenous Great Lakes salmonid species [3]. Exacerbating the negative effects of this virus is the complete lack of efficacious vaccine or treatment options for EEDV-infected lake trout stocks, limiting EEDV prevention and control measures to avoidance, culling, and/or depopulation.

Hatchery biosecurity, defined generally as a set of preventative measures designed to reduce the risk of introducing infectious agents into a facility, prevent pathogen transmission within a facility, and avert dissemination to neighboring facilities/the environment [4], is a cornerstone of disease prevention. Among the multitude of means by which infectious agents can enter and/or spread within hatcheries and aquaculture facilities is mechanical transport via pathogen-contaminated fomites (e.g., nets, boots, brushes, transport vessels, etc.). Indeed, several highly pathogenic fish viruses persist and remain infectious on inanimate surfaces for durations that facilitate contagion to new hosts [5,6], including some fish herpesviruses [7]. Although previous work with EEDV has demonstrated that water is an important transmission vehicle [8], the role that contaminated fomites may play in contagion has yet to be adequately explored, a matter of concern for fishery managers attempting to devise EEDV control and prevention plans. 

Hatchery disinfectants are frequently utilized to not only prevent the introduction of fish pathogens into hatchery systems via contaminated fomites, but also to limit their spread within hatcheries themselves [9]. Virkon^®^ Aquatic (Syndel, Ferndale, Washington) is a potassium peroxymonosulfate (PPMS)-based disinfectant that has viricidal and bactericidal effects [10], is relatively safe for fish [11], and is therefore commonly used in commercial aquaculture facilities to disinfect fish husbandry tools [12]. PPMS-based disinfectants are efficacious at inactivating a range of viruses [13,14,15,16]; however, the efficacy of Virkon^®^ Aquatic has yet to be determined against herpesviruses that infect fish, including EEDV. Therefore, this study was designed to not only investigate the potential role of fomites in EEDV transmission, but also to assess the efficacy of Virkon^®^ Aquatic for preventing or minimizing EEDV transmission, thereby providing hatchery managers with scientific evidence to assist in the establishment of proper biosecurity measures against EEDV.

## 2. Results

### 2.1. EEDV Loads in Experimental Inocula and Net Treatment Solutions

The estimated EEDV loads in the virus suspensions prior to net soaking ranged from 0–2.25 × 10^5^ virus copies/mL suspension, and from 0–1.77 × 10^5^ virus copies/mL suspension after net soaking (Table 1). No EEDV was detected in the negative control Earle’s salt-based minimal essential medium (EMEM; Invitrogen, Thermo Fisher Scientific, Waltham, Massachusetts) solutions before or after net soaking. These results are detailed in Table 1.

### 2.2. Gross Disease Signs and Cumulative Mortality

**Figure 1:**
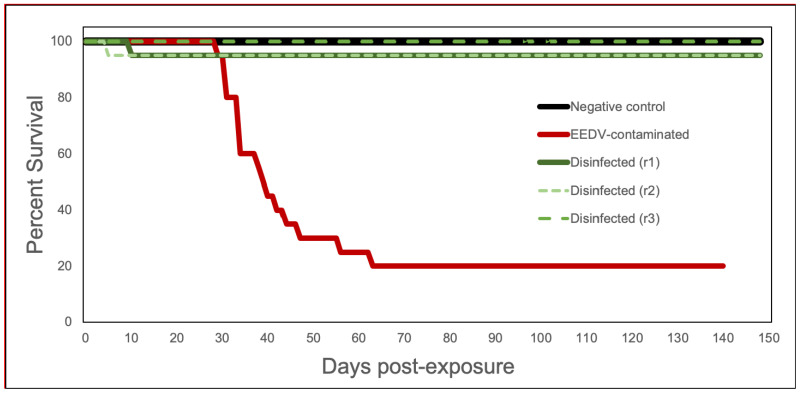
Survival curves of lake trout exposed to Epizootic epitheliotropic disease virus (EEDV) via contaminated nets, with and without disinfection with Virkon Aquatic^®^. Negative control (black), EEDV-contaminated (red) and three replicates of disinfection (green; designated r1, r2, and r3).

The mean length of lake trout at the time of necropsy was 18.2 cm (standard deviation = 3.0), with a mean weight of 54.9 g (standard deviation = 27.1). Behavioral changes and gross disease signs consistent with clinical EED were observed in the EEDV-contaminated group beginning at day 26 post-infection (pi) and included lethargy, unilateral to bilateral exophthalmia, and corneal opacity. As the disease progressed, ocular hemorrhage, severe congestion at fin bases, focal to multifocal opacity and thickening of the skin, and secondary water mold invasion of the fins, body, and eyes were observed. The first mortality in the EEDV-contaminated group occurred on day 29 pi, with subsequent mortalities continuing until day 63 pi, ultimately reaching 80% cumulative mortality (Figure 1). In contrast, no disease signs consistent with EED were observed in any fish within the three disinfected treatment replicates. One fish died in two of the three disinfected treatment replicates, both of which were attributed to aggression (Table 2; Figure 1). No mortality or EED disease signs were observed in any negative control fish throughout the course of this study. Using a one-way ANOVA, and a Tukey–Kramer post hoc test, the cumulative percent mortality (CPM) of the negative control and EEDV-contaminated group were significantly different (*p* = 0.003; q = 27.7), as were the CPM of the Virkon^®^ Aquatic treatment groups and the EEDV-contaminated group (*p* = 0.003; q = 32.5). However, the CPM of the negative control group and the Virkon^®^ Aquatic treatment groups were not significantly different from one another (q = 1.41).

### 2.3. Molecular Detection of EEDV

EEDV was not detected in any of the NC fish, nor in any fish within the disinfected groups (Table 2). In the EEDV-contaminated group, however, 18/20 fish were EEDV-positive via qPCR, with calculated viral loads exceeding the initial challenge concentration (7.29 × 10^4^ virus copies/mL of water) and ranging from 2.16 × 10^7^ to 7.58 × 10^8^ virus copies/mg skin tissue (mean: 2.11 × 10^8^; median: 1.39 × 10^8^) in fish that died (Table 2). Among the EEDV-positive individuals, 16 died and 2 were euthanized at 140 days pi. The two EEDV-negative fish did not show any gross signs of disease at the time of euthanasia (140 days pi).

## 3. Discussion

Contaminated equipment has been implicated in the transmission of numerous microbial fish pathogens [17]. Fortunately, fomite disinfection is one of several tools that can successfully prevent pathogen transmission into, within, and out of a hatchery system. Prior to the current study, however, it was unknown whether hatchery equipment could serve as a passive carrier (i.e., fomite) for EEDV specifically. Additionally, the efficacy of hatchery disinfectants against EEDV was unknown, further hampering the design of hatchery biosecurity protocols aimed at preventing EEDV transmission. This study definitively demonstrates that contaminated nets can serve as a fomite for EEDV transmission. The knowledge that EEDV can be transmitted via contaminated hatchery equipment underscores the importance of implementing the strict disinfection of all hatchery utensils and tools. 

Study findings also provide evidence that under laboratory conditions and when used following the manufacturer’s directions, Virkon^®^ Aquatic can prevent EEDV losses via a contaminated net to a highly susceptible host species (i.e., a Lake Superior strain lake trout). Indeed, despite the development of the characteristic and severe EED (e.g., high mortality, infection prevalence, and virus load) in lake trout netted with an EEDV-contaminated net, no signs of disease, EED-associated mortality, or the virus itself were detected in fish handled with an EEDV-contaminated net that was treated with 1% Virkon^®^ Aquatic.

Although this study was the first to empirically assess EEDV susceptibility to Virkon^®^ Aquatic, previous studies have examined the efficacy of Virkon^®^ against other enveloped double stranded DNA viruses, including hepatitis B [15,18], a Ranavirus [19], and adenovirus 5 and 6 [20]. Specifically concerning herpesviruses, Tsujimura et al. [16] found that Virkon^®^ effectively inactivated Equine Herpesvirus-1 (family *Herpesviridae*) and Hick et al. [21] reported that it inactivated Ostreid Herpesvirus-1 (family *Malacoherpesviridae*). The current study demonstrates that Virkon^®^ Aquatic can be used to prevent transmission of EEDV, a fish-pathogenic herpesvirus (family *Alloherpesviridae*).

One of the limitations of the current study, primarily brought about by the inability to culture EEDV in vitro, was the relatively low EEDV concentration that was utilized to contaminate the experimental nets. Despite this, the utilized virus concentration in the net soak suspension (i.e., 7.29 × 10^4^–2.25 × 10^5^ virus copies/mL) exceeded the estimated median lethal dose for EEDV via immersion (i.e., 4.7 × 10^4^ virus copies/mL; [22]) and, although less than the maximum load of infected fish have been shown to shed (i.e., up to 2.47 × 10^8^ virus copies/fish/hour; [8]), nevertheless led to subsequent EEDV infection in 90% of exposed fish with evidence of virus replication (i.e., virus loads in tissues in excess of the original exposure dose). Likewise, the current experimental challenge model led to initial mortality (day 29 pi) that was similar to what was observed via immersion challenge by Shavalier et al. [22] (day 28 pi) and thus shows promise for future disinfection studies that aim to mimic common hatchery practices. Although this potentially low virus dose lends support to the ability of nets to act as fomites, it remains unknown whether Virkon^®^ Aquatic would be as efficacious against higher viral loads. 

Although not the focus of this study, it is of interest to note that of the four-lake trout that survived to 140-day pi, two still harbored high EEDV loads; unfortunately, a data curation failure prevented virus loads from being estimated in these two fish. In comparison, another study [23] demonstrated the EEDV viral loads ranging from 1.33 × 10^4^–5.83 × 10^6^ viral copies/mg skin in surviving Lake Superior strain lake trout at the end of a 66-day experimental challenge, and 1.59 × 10^7^–7.18 × 10^7^ virus copies/mg skin in Seneca Lake strain lake trout at 100 days pi. Although the route of virus exposure varied between the current study and previous challenges (i.e., net exposure vs. intracelomic injection), both demonstrated that lake trout can harbor EEDV in their skin for extended periods of time. This, combined with the ability of nets to act as fomites, increases the risk of virus transmission between infected and naïve populations. 

In conclusion, herein we provide the first definitive evidence that EEDV can be transmitted via fomites, and that fomite disinfection with Virkon^®^ Aquatic is a promising means of reducing the risk of EEDV contagion on contaminated hatchery equipment. Although Virkon^®^ Aquatic is marketed for effective disinfection in the presence of organic material, further studies should focus on evaluating its capacity to prevent EEDV transmission through hatchery equipment under field conditions.

## 4. Materials and Methods

### 4.1. Experimental Challenge via EEDV-Contaminated Fomites

#### 4.1.1. Fish and Husbandry

Four-month-old Lake Superior strain lake trout maintained on a closed (i.e., deep well) water source were obtained from Marquette State Fish Hatchery (MSFH; Marquette, MI, USA). Upon arrival at the Michigan State University—University Research Containment Facility (URCF), and until the experimental challenge (at 22 months of age), fish were housed in a 680 L flow-through fiberglass tank supplied with ultraviolet-irradiated, oxygenated deep well water (11–14 °C) and fed AquaMax^®^ Fingerling Starter 300 (Purina^®^, Gray Summit, MO, USA) until satiation, with detritus/feces siphoned and removed daily. A subset of the fish was examined for the presence of EEDV via qPCR (see below) to ensure freedom from infection prior to use in experimental challenges. All fish handling and maintenance during the study was performed in accordance with the Institutional Animal Care and Use Committee (IACUC) standards (AUF #11/17-197-00).

#### 4.1.2. Preparation of EEDV Inoculum

As EEDV has yet to be cultured in vitro, a homogenate containing infectious virus was prepared from skin collected from experimentally induced, EEDV-infected lake trout as previously described [22]. The skin was manually trimmed to approximately 1–2 mm sections, and homogenized in a sterile sample diluent (pH 7.525 ± 0.025) of Earle’s salt-based minimal essential medium (EMEM; Invitrogen, Thermo Fisher Scientific, Waltham, MA, USA) supplemented with 10% BD Bacto^TM^ tryptose phosphate broth (Becton, Dickinson and Company, Sparks, MD, USA), 12 mM tris buffer (Sigma-Aldrich, St. Louis, MI, USA), 0.1 mg/mL gentamycin sulfate (Sigma-Aldrich), 100 IU/mL penicillin (Invitrogen), 100 µg/mL streptomycin (Invitrogen), and 2.5 µg/mL Amphotericin B (Thermo Fisher Scientific) at a 1:3 ratio (*w*/*v*). The homogenate was then centrifuged at 368× *g* for 20 min at 4 °C, and aliquots of the supernatant were frozen at −80 °C for use in the challenge. 

#### 4.1.3. Disinfectant Solution Preparation

One hour prior to the infection challenge, a 1% Virkon^®^ Aquatic solution (manufacturer’s recommended concentration for equipment disinfection) was prepared following the manufacturer’s protocol. In brief, 8.5 g of Virkon^®^ Aquatic was dissolved into 946 mL (1 quart) clean tank water. Then, 600 mL of this solution was transferred into an 11.4 L glass aquarium. A fresh batch of Virkon^®^ Aquatic was prepared for each experimental replicate.

#### 4.1.4. Experimental Challenge of Juvenile Lake Trout with EEDV

Prior to the EEDV challenge, fish were randomly divided into five flow-through 42-L fiberglass cylindrical tanks filled to 18.9 L and acclimated to chilled (10 ± 1.0 °C) water over 15 days. The five experimental tanks were categorized into three treatment groups: negative control (NC; 1 tank, n = 20 fish), disinfected (1% Virkon^®^ Aquatic; 3 tanks, n = 20 fish/tank), and EEDV-contaminated (EEDV; 1 tank, n = 20 fish).

**Figure 2:**
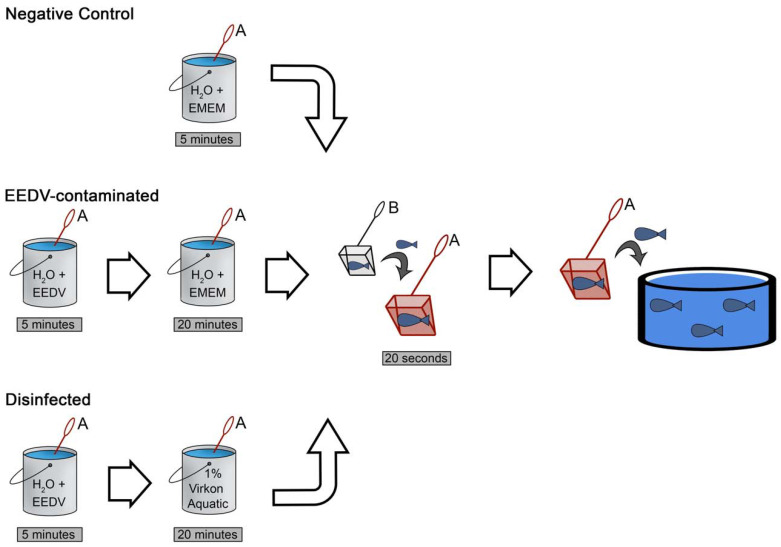
Diagram depicting the steps of the EEDV challenge and net disinfection experiment. NetA was contaminated/disinfected; NetB was for fish transfer only. All treatment groups utilized 20 fish each. The disinfected (1% Virkon^®^ Aquatic) treatment was repeated in triplicate. EMEM, Earle’s salt-based minimal essential medium; EEDV, epizootic epitheliotropic disease virus.

All challenges began with specific treatments of Net_A_ (detailed below; Figure 2). After Net_A_ treatments were completed, a second net (Net_B_) was used to transfer all 20 fish from their experimental tank into Net_A_, where they were held for 20 s and then transferred immediately back to their flow-through experimental tank. All treatments and replicates utilized separate nets and fresh batches of EMEM and disinfectant solutions. A single EEDV solution was used for all “contamination” steps. The Net_A_ treatments for the three treatment groups were as follows (Figure 2):

1. Negative control (NC): Net_A_ was soaked in 600 mL tank water containing 7 mL sterile EMEM for 5 min;
2. Disinfected (Virkon^®^ Aquatic): Net_A_ was soaked in 600 mL water containing 7 mL of EEDV homogenate (final concentration of 2.25 × 10^5^ virus copies/mL of water) for 5 min. Immediately following, Net_A_ was soaked in 600 mL of 1% Virkon^®^ Aquatic solution for 20 min (manufacturer recommended duration);
3. EEDV-contaminated: Net_A_ was soaked in 600 mL water containing 7 mL of EEDV homogenate (final concentration of 7.29 × 10^4^ virus copies/mL of water) for 5 min. Immediately following, Net_A_ was soaked in 600 mL of 1% EMEM: water solution for 20 min.

Following experimental infection, all fish were observed daily for the development of disease signs or mortality, fed to satiation twice daily with AquaMax^®^ Fingerling Starter 300, and tanks were cleaned as described above. Any terminally moribund fish were euthanized via a lethal dose (0.25 mg/mL) of tricaine methanesulfonate (MS-222; Western Chemical Inc., Ferndale, WA, USA) buffered with 0.5 mg/mL of sodium bicarbonate (Church & Dwight Co., Inc., Ewing, NJ, USA). After death or euthanasia, the weight and length of each fish was measured and then fish were clinically examined, necropsied, and skin tissue was collected as previously described [22], before they were frozen individually at −20 °C. At the end of the experiment (140 days post-infection for the EEDV-contaminated group, 148 days post-infection for disinfected and NC groups), all surviving fish were euthanized with buffered MS-222 and examined/processed in the same fashion.

#### 4.1.5. Water and Suspension Sampling

To assess EEDV loads throughout the exposure and disinfection process, samples were collected from the following sources: (1) negative control suspension prior to net soaking; (2) negative control suspension after net soaking; (3) EEDV suspension (prior to net soaking); (4) disinfected group Virkon^®^ Aquatic suspension after net soaking; and (5) EEDV-contaminated group EMEM:water suspension after net soaking. EEDV loads were assessed using quantitative PCR as detailed below.

### 4.2. Molecular Detection and Quantification of EEDV

#### 4.2.1. DNA Extraction

Skin tissues were thawed and trimmed so that a maximum of 10 mg of tissue could be transferred into a sterile 1.5 mL tube for DNA extraction. All tissue extractions were performed using the Mag-Bind^®^ Blood & Tissue DNA Kit (OMEGA Bio-tek, Inc, Norcross, Georgia) following the manufacturer’s protocol with the addition of filtering the digested tissue through an E-Z 96^®^ Lysate Clearance Plate (OMEGA Bio-tek, Inc.) prior to extraction. DNA extraction from water and solution samples was performed using the Qiagen DNeasy^®^ PowerLyzer^®^ PowerSoil^®^ Kit (Qiagen, Hilden, Germany) with some modifications to improve yield from low biomass fluids as previously described [8]. Extracted DNA from both protocols was quantified using a Qubit™ fluorometer (Invitrogen, Eugene, OR, USA) and Qubit™ dsDNA BR Assay Kit following the manufacturer’s protocol, and samples were diluted with sterile DNase-free water to obtain a maximum of 12.5 ng/μL template DNA for qPCR.

#### 4.2.2. Quantitative PCR Analysis

The SYBR Green qPCR assay, described by Glenney et al. [24], which targets a portion of the EEDV glycoprotein gene, was utilized in this study. All qPCR reactions (20 µL) were carried out in a Mastercycler ep realplex2 real-time PCR machine (Eppendorf, Hauppauge, New York, NY, USA). Each reaction contained 10 µL SYBR Select Master Mix (2×; Life Technologies), 1.0 µM of forward and reverse primers and 50 nmol total DNA template. Extraction controls consisted of EEDV-positive tissue homogenate (positive extraction control, PEC) and EMEM (negative extraction control, NEC). PCR controls included EEDV-positive purified DNA (positive amplification control) and nuclease-free water (negative amplification control). Samples were considered EEDV-positive if the fluorescence exceeded 10% of the maximum florescence within 35 amplification cycles. Viral loads (copies/mg and copies/mL) were calculated using the Mastercycler ep realplex2 S accompanying software via comparison to a standard curve that was generated via 8 serial 10-fold dilutions of EEDV-positive standards [22].

### 4.3. Statistical Analysis

Cumulative percent mortality (CPM) of the treatment groups (i.e., negative control, EEDV-contaminated, disinfected) was analyzed by one-way ANOVA. Pairwise comparisons were made using the Tukey–Kramer test. All analyses were conducted in Microsoft Excel [25], and α was set at 0.05.

## Figures and Tables

**Table 1:** Epizootic epitheliotropic disease virus (EEDV) loads in experimental suspensions and net treatment solutions. EMEM: Earle’s salt-based minimal essential medium.

Suspension	Virus Load (Virus Copies/mL)
Virus suspension prior to net soaking (EEDV-contaminated group)	7.29 × 10^4^
Virus suspension prior to net soaking (disinfected groups)	2.25 × 10^5^
EMEM solution prior to net soaking (negative control group)	0
EMEM after net soaking (EEDV-contaminated group)	1.77 × 10^5^
1% Virkon^®^ Aquatic after net soaking (disinfected groups)	1.85 × 10^4^
EMEM after net soaking (negative control group)	0

**Table 2:** Lake trout mortalities and PCR results following a challenge with Epizootic epitheliotropic disease virus (EEDV) via a contaminated net with and without disinfection by 1% Virkon^®^ Aquatic.

Challenge Group	Mortalities	Number PCR-Positive	EEDV Viral Load Range (Copies/mg)
Negative control	0/20	0/20	0
EEDV-contaminated	16/20	18/20	2.16 × 10^7^–7.58 × 10^8^ *
Disinfected			
Replicate 1	1/20	0/20	0
Replicate 2	1/20	0/20	0
Replicate 3	0/20	0/20	0

* EEDV viral load in EEDV-contaminated group is that of 16 mortalities only.

## Data Availability

The data presented in this study are available on request from the corresponding author.
